# Risk-Benefit of IBD Drugs: A Physicians and Patients Survey

**DOI:** 10.3390/jcm12093094

**Published:** 2023-04-24

**Authors:** Ferdinando D’Amico, Sophie Vieujean, Benedicte Caron, Laurent Peyrin-Biroulet, Silvio Danese

**Affiliations:** 1 Department of Gastroenterology and Endoscopy, IRCCS San Raffaele Hospital, Vita-Salute San Raffaele University, 20132 Milan, Italy; 2 Department of Biomedical Sciences, Humanitas University, Pieve Emanuele, 20090 Milan, Italy; 3 Hepato-Gastroenterology and Digestive Oncology, University Hospital CHU of Liège, 4000 Liège, Belgium; s.vieujean@chuliege.be; 4 INSERM, NGERE, University of Lorraine, F-54000 Nancy, France; 5 Department of Gastroenterology, Nancy University Hospital, F-54500 Vandœuvre-lès-Nancy, France

**Keywords:** benefit-risk, inflammatory bowel disease, patients, physicians

## Abstract

Background: Treatment choices for patients with inflammatory bowel disease (IBD) are based on the balance between risks and benefits. Our AI was to compare the perspectives of patients and physicians in evaluating the risks and benefits before initiating therapy for IBD. Methods: An anonymous survey was conducted between March and August 2022. All patients with confirmed IBD and all physicians who attended an IBDscope webinar were invited to participate. Results: In total, 367 patients and 146 physicians participated. For most patients (71.4%) and physicians (89.0%), efficacy and safety were equally important. Clinical improvement and clinical remission were the most relevant outcomes for patients (90.9 and 88.4), while clinical remission and endoscopic remission were for physicians (90.0 and 87.6). The main factors in the benefit-risk assessment were quality of life (95.1%), disease activity (87.5%), and presence of comorbidities (84.5%) for patients, and presence of comorbidities (99.3%), disease activity (97.9%), and prior failure to biologics/small molecules (96.6%) for physicians. Based on patients’ and physicians’ opinions, the risk of serious infections, malignancies, cardiovascular events, death, relapse, all infections, surgery, and hospitalization should be included in the benefit-risk assessment. Conclusion: Physicians and patients have different priorities in evaluating the benefit-risk balance of a new therapy.

## 1. Introduction

Crohn’s disease (CD) and ulcerative colitis (UC) are inflammatory bowel diseases (IBDs) with a remitting and relapsing course [1,2]. They are chronic diseases with a significant burden on patients’ quality of life. Over the past twenty years, the therapeutic approach in IBD has changed considerably. In addition to conventional therapies (e.g., aminosalicylates, steroids, and immunosuppressants), several biological drugs and small molecules are available, expanding the therapeutic armamentarium [3,4]. On the other hand, even the treatment targets have evolved, passing from symptom control to clinical and endoscopic remission, with the prospect of achieving increasingly profound remission goals such as histological remission in UC and transmural healing in CD [5]. Despite the extraordinary development in recent years, the currently available options have an efficacy that does not exceed 60% of cases [6]. Several strategies have been proposed to break this therapeutic ceiling, including the development of more effective drugs, early therapies, and new drug combination strategies. However, the attempt to have increasingly effective treatments must be balanced with the safety profile of the drugs. Of note, each therapeutic choice is based on the risk-benefit ratio associated with a specific drug. Although the risk-benefit ratio plays a key role, to date there is no globally accepted tool for measuring this fundamental parameter [7]. Moreover, there is no agreement on what is considered important by physicians and patients, making the definition of the risk-benefit ratio even more complex. For this reason, we developed a survey with the aim to investigate the perspectives of patients and physicians in evaluating the risks and benefits before initiating a new therapy for IBD.

## 2. Materials and Methods

### 2.1. Study Design

An anonymous survey was conducted between March and August 2022. All patients with a confirmed diagnosis of IBD who were followed up at the IRCCS San Raffaele Hospital (Italy), Nancy University Hospital (France), and University Hospital CHU of Liège (Belgium) were invited to participate. All physicians who attended an IBDscope live webinar were invited to participate by email. The email address used to register for the online event was used to send invitations for the survey.

### 2.2. Questionnaire and Outcomes

The survey consisted of multiple-choice questions, similar for physicians and patients, evaluating the relevance of the efficacy and safety data and the parameters to be considered before choosing a therapy (e.g., age, comorbidities, disease location, family history of IBD, and previous failed therapies) (Supplementary Files S1 and S2). Furthermore, demographic characteristics of patients (e.g., age, type of disease, and prior therapy) and physicians (e.g., specialty, number of IBD patients followed up per year, and years of experience in the field of IBD) were collected. Both physicians and patients were also asked to rate the importance of individual factors tested (e.g., achievement of clinical response, clinical remission, endoscopic improvement, or endoscopic remission) using a scale that ranged from 0 (lowest value) to 100 (highest value). Additionally, physicians were asked to rate the efficacy and safety of currently available drugs for the induction and maintenance of remission in CD and UC using a scale ranging from 0 (lowest value) to 100 (highest value). A value greater than 75 was considered high, a value between 50 and 75 was of moderate importance, and a value less than 50 was considered insignificant. Three physicians (FD, LPB and SD) created the survey questions in English. Subsequently, the questionnaire was back-translated into Italian and French by native speakers.

### 2.3. Ethics Approval

The survey was non-interventional, and it was not intended to provide clinical data for treatment decisions. It was not conducted as a clinical trial for any endpoints; in Italy, France, and Belgium ethics approval is not required for non-interventional surveys of patients.

## 3. Results

### 3.1. Patients’ Survey

#### 3.1.1. Patients’ Demographic

In total, 367 consecutive patients (163 male, 44.4%) with a mean age of 42.2 ± 14.6 years participated in the survey (Table 1). Most patients had a diagnosis of CD (221, 60.2%), while a lower number of subjects had UC (144, 39.2%) or unclassified IBD (2, 0.6%). More than half of the respondents were Italian (203, 55.3%) while the rest were from France (140, 38.1%) and Belgium (24, 6.5%). Mean age at diagnosis of IBD was 29.7 ± 13.3 years. Most patients were previously treated with biological drugs and small molecules (298, 81.2%), steroids (256, 69.7%), and immunosuppressants (163, 44.4%).

#### 3.1.2. Factors to Consider before Starting a New Therapy

For about three-quarters of patients (262, 71,4%), efficacy and safety were equally important in the benefit-risk assessment before starting a new therapy. Interestingly, only one in five patients thought efficacy was the most important factor (80, 21.8%), while only a small percentage of patients prioritized the drug’s safety (25, 6.8%). Drug efficacy achieved a mean relevance of 77.7 ± 20.4 points, while the safety profile had a mean value of 71.9 ± 29.4 points. Of note, the prevailing opinion among participants (306, 83.4%) was that in patients at risk of adverse events (e.g., pediatric patients, pregnant women, the elderly, and personal cancer history), the safety profile should be considered the most important parameter in drug selection.

#### 3.1.3. Efficacy Outcomes

According to most patients, the possibility of achieving clinical improvement (359, 97.8%), clinical remission (334, 91.0%), endoscopic improvement (324, 88.3%), and endoscopic remission (316, 86.1%) should be included in the evaluation of the benefit-risk ratio of a new therapy. Biochemical remission (315, 85.8%) and histological remission (299, 81.5%) should also be considered, while about one third of patients did not consider radiological remission to be important (35, 9.5%) or did not know its usefulness (95, 25.9%). The most relevant outcomes to achieve for patients were clinical improvement (90.9 ± 13.1), clinical remission (88.4 ± 16.6), endoscopic remission (85.0 ± 20.2) and endoscopic improvement (84.8 ± 19.5), followed by histologic remission (83.4 ± 20.5), biochemical remission (81.1 ± 20.6), and radiological remission (75.1 ± 23.7).

#### 3.1.4. Patients’ Characteristics Associated with the Risk-Benefit Ratio of a New Therapy

In almost all cases (349, 95.1%), patients’ quality of life was a relevant factor for the therapeutic choice of a drug. Similarly, activity of disease (321, 87.5%), presence of comorbidities (310, 84.5%), and disease extension (295, 80.4%) were recognized as relevant. Approximately three-quarters of patients (279, 76.0%) felt that the previous failure of a biologic drug or a small molecule should be considered. Moreover, response time to therapy was a relevant factor for most respondents (260, 70.8%). In about two-thirds of cases, a response time <4 weeks (101/260, 38.9%) or <3 months (64/269, 24.6%) was considered acceptable to justify the use of a drug. Age (254, 69.2%) and disease duration (251, 68.4%) were also considered important for most patients. Conversely, other factors such as administration route (167, 45.5%), therapy duration (148, 40.3%), administration interval (147, 40.1%), family history of IBD (145, 39.5%), and need to go to the hospital 131 (35.7%), had a limited role or an unknown importance.

#### 3.1.5. Safety Outcomes

**Figure 1:**
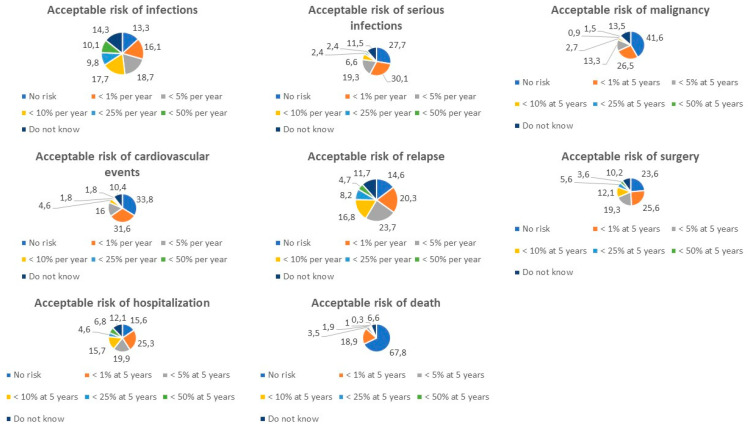
Percentages of risk considered acceptable by patients.

The risk of infections (316, 86.1%) and serious infections (332, 90.5%) were key aspects to consider before starting a new therapy. Of note, the acceptable risk of infections per year was highly variable, ranging from no risk (42/316, 13.3%), to <1% (51, 16.1%), <5% (59, 18.7%), <10% (56, 17.7%), <25% (31, 9.8%), and <50% (32, 10.1%), or unknown (45, 14.3%) (Figure 1). On the other hand, most of the respondents accepted no risk of serious infections (92/332, 27.7%), a risk of <1% per year (100, 30.1%), or <5% per year (64, 19.3%). The risk of malignancy (332, 90.5%), cardiovascular events (326, 88.8%), death (317, 86.4%), disease relapse (316, 86.1%), surgery (305, 83.1%), and hospitalization (281, 76.6%) also needed to be assessed for the majority of patients. For most subjects (138/332, 41.6%) a new therapy had to be associated with no risk of malignancy. However, approximately one-quarter of patients (88, 26.5%) considered a 5-year risk of < 5% to be acceptable, while a lower percentage of subjects were willing to accept a risk of <5% at 5 years (44. 13.3%), <10% at 5 years (9, 2.7%), <25% at 5 years (3, 0.9%), or <50% at 5 years (5, 1.5%). The risk of cardiovascular events was unacceptable in one-third of cases (110/326, 33.8%), but about half of the patients considered a risk of <1% per year (103, 31.6%) or <5% per year (52, 16.0%) to be acceptable. The tolerable risk for hospitalization and surgery was highly variable with most patients accepting a risk of <1% at 5 years (71/281, 25.3% and 78/305, 25.6%, respectively). Similarly, there was no agreement on the risk of disease relapse, but the majority tolerated a risk of <5% per year (75, 23.7%). Finally, the risk of death was unacceptable for two-thirds of the patients (215/317, 67.8%), but surprisingly one-fifth of the patients (60, 18.9%) considered a risk of <1% at 5 years as valid and a limited number of patients accepted even a higher risk (21, 6.7%).

### 3.2. Physicians’ Survey

#### 3.2.1. Physicians’ Demographic

In total, 146 out of 627 invited (23.3%) physicians (78 male, 53.4%) with a mean age of 51.8 ± 11.3 years from 34 countries worldwide participated in the survey (Table 2). Italy (22, 15.1%), Brazil (8, 5.5%), and Lebanon (8, 5.5%) were the most represented countries, followed by Russia (7, 4.8%), Australia (6, 4.1%), and Greece (6, 4.1%) (Supplementary File S3). Most physicians specialized in gastroenterology (129, 88.4%), but also internal doctors (6, 4.1%), surgeons (5, 3.4%), other specialists (5, 3.4%), and one general practitioner (0.7%) were involved. Over two-thirds of respondents (105, 71.9%) had more than 10 years of experience in the field of IBD, but most of them visited <100 IBD patients per year (70, 48.0%) or 100–500 IBD patients per year (41, 28.1%).

#### 3.2.2. Factors to Consider before Starting a New Therapy

Almost all physicians (130, 89.0%) believed that efficacy and safety were equally important in the benefit-risk assessment before starting a new therapy. Only a small percentage of physicians prioritized efficacy (12, 8.2%) or safety (4, 2.8%). The mean relevance of drug efficacy and safety in evaluating the benefit-risk ratio of IBD therapy was higher than the patient-reported value with 87.4 ± 13.9 points and 89.7 ± 11.7 points, respectively. Again, for most of the respondents (135, 92.5%), in patients at risk of adverse events (e.g., pediatric patients, pregnant women, the elderly, and personal cancer history), the safety profile should be considered the most important parameter in the therapeutic decision.

#### 3.2.3. Efficacy Outcomes

According to the majority of physicians, the possibility of achieving clinical remission (146, 100.0%), endoscopic response (142, 97.3%), endoscopic remission (142, 97.2%), biochemical remission (140, 95.9%), and clinical response (139, 95.2%), should be included in the evaluation of the benefit-risk ratio of a new therapy. Histological remission (130, 89.0%) and radiological remission (130 89.0%) were also considered relevant. The most consistent outcomes to achieve for physicians were clinical remission (90.0 ± 12.5), clinical response (88.0 ± 12.8), endoscopic remission (87.6 ± 13.4) and endoscopic response (87.6 ± 13.7), followed by biochemical remission (83.3 ± 14.5), histologic remission (79.0 ± 18.2), and radiological remission (75.8 ± 19.6).

#### 3.2.4. Patients’ Characteristics Associated with the Risk-Benefit Ratio of a New Therapy

For almost all physicians (145, 99.3%), presence of comorbidities had to be evaluated before starting a new therapy. Activity of disease (143, 97.9%), prior failure towards biologics or small molecules (141, 96.6%), patients’ quality of life (138, 94.5%), patient age (136, 93.1%), and disease extension (132, 90.4%) were relevant factors for the majority of physicians. Response time to therapy also played a major role (138, 94.5%) and most respondents believed that a response within 3 months (61/138, 44.2%) or within 2 weeks (54, 39.1%) were acceptable to justify the use of a drug. More than three-quarters of physicians considered need to go to hospital (127, 87.0%), disease duration (122, 83.6%), administration interval (116, 79.5%), and therapy duration (111, 76.0%) to be important. On the other hand, familiarity with IBD (65, 44.5%) and drug administration route (43, 29.5%) played a marginal role for a fair percentage of doctors.

#### 3.2.5. Safety Outcomes

**Figure 2:**
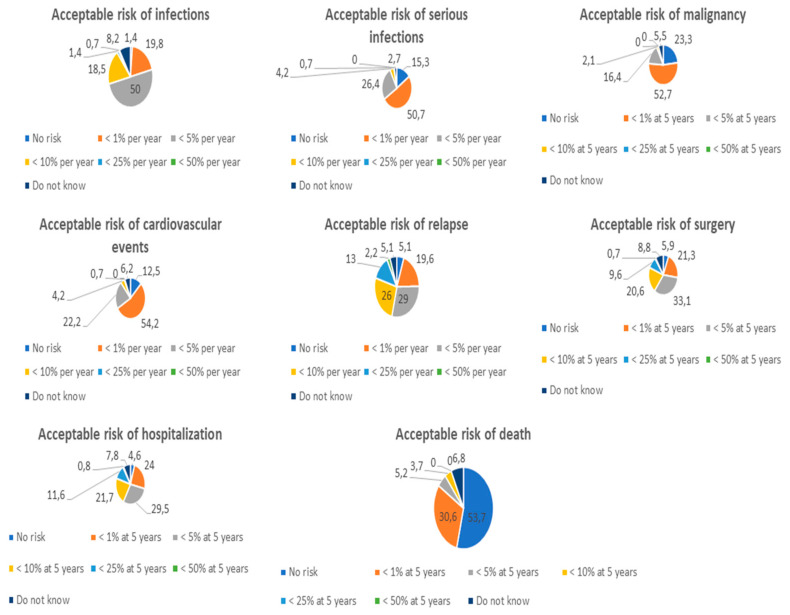
Percentages of risk considered acceptable by physicians.

According to all participants, the risk of infections (146, 100.0%) and malignancy (146, 100.0%) had to be included in the benefit-risk assessment of an IBD patient who is a candidate for therapy. An infectious risk of <5% per year was acceptable to half of the physicians (73, 50.7%), but a risk of <1% per year (29, 19.8%) or <10% per year (27, 18.5%) was also deemed appropriate (Figure 2). As regards the risk of malignancy, a risk of <1% at 5 years was acceptable for half of the respondents (77, 52.7%), while a quarter of the doctors (34, 23.3%) did not consider it acceptable at all. In addition, the risks of cardiovascular events (144, 98.6%), serious infections (144, 98.6%), disease recurrence (138, 94.5%), surgery (136, 93.1%), death (134, 91.8%), and hospitalization (129, 88.4%) had to be assessed according to almost all physicians. The acceptable risk of cardiovascular events and serious infections had similar trends with about half of the subjects accepting a risk of <1% per year (78/144, 54.2% and 73/144, 50.7%), a quarter considering a risk of <5% per year tolerable (32/144, 22.2% and 38/144, 26.4%), and a small percentage of subjects accepting no risk (18/144, 12.5% and 22/144, 15.3%). Similarly, the trends in surgery and hospitalization risks were equivalent, with most subjects accepting a risk of <5% at 5 years (45/136, 33.1% and 38/129, 29.5%), followed by a risk of <1% at 5 years (29, 21.3% and 31, 24.0%) and <10% at 5 years (28, 20.6% and 28, 21.7%). There was high variability in the risk of disease recurrence with approximately one-third of respondents accepting a risk of <5% per year (40/138, 29.0%) followed by <10% per year (36, 26.0%), <1% per year (27, 19.6%), and <25% per year arms (18, 13.0%). Finally, no risk of death should be associated with a new drug for about half of the doctors (72/134, 53.7%), while a risk of <1% at 5 years was tolerable for one-third of the respondents (41, 30.6%). Only a limited percentage of physicians considered acceptable a risk of death of <5% at 5 years (7, 5.2%) or <10% at 5 years (5, 3.7%).

#### 3.2.6. Efficacy and Safety of Drugs in Inducing and Maintaining Remission

Infliximab was ranked as the most effective drug in inducing (82.5 ± 11.5 and 83.7 ± 10.7) and maintaining (78.8 ± 12.6 and 80 ± 12.2) disease remission in both CD and UC. After infliximab, the best drugs to induce remission in UC were vedolizumab (75.6 ± 14.4), tofacitinib (75.3 ± 16.3), and ustekinumab (74.8 ± 16.9), followed by adalimumab (71.6 ± 17.1) and golimumab (64.5 ± 20.0). Regarding the maintenance of remission in UC, vedolizumab (78.9 ± 12.6), ustekinumab (75.3 ± 17.2), and tofacitinib (74.5 ± 15.9) were the most effective drugs after infliximab, whereas adalimumab (72.8 ± 17.3) and golimumab (64.0 ± 18.8) had lower efficacy. In CD, efficacy rankings for both induction and maintenance phases had ustekinumab immediately following infliximab (78.7 ± 14.9 and 78.3 ± 15.6) and then adalimumab (77.8 ± 12.6 and 76.7 ± 14.1), and vedolizumab (72.1 ± 15.4 and 73.2 ± 16.6). Interestingly, vedolizumab (82.6 ± 14.8) and ustekinumab (82.6 ± 13.7) were equally regarded as the drugs with the best safety profile, followed by adalimumab (77.7 ± 12.3), infliximab (77.1 ± 13.2), tofacitinib (68.6 ± 17.8), and golimumab (64.2 ± 18.7). Thiopurines and methotrexate were considered drugs with limited efficacy in inducing (51.7 ± 27.4 and 51.7 ± 29.0 for thiopurines in CD and UC; 48.5 ± 22.2 and 35.4 ± 29.1 for methotrexate in CD and UC) and maintaining (51.7 ± 18.1 and 51.7 ± 18.2 for thiopurines in CD and UC; 53.9 ± 18.5 and 37.0 ± 28.0 for methotrexate in CD and UC) remission. Similarly, the overall safety profile of thiopurines (63.0 ± 17.7) and methotrexate (54.9 ± 18.9) was also suboptimal.

## 4. Discussion

Our survey investigates the parameters to be evaluated before starting a therapy for IBD, focusing on both patients’ and physicians’ point of view. Both physicians and patients agreed that efficacy and safety were equally important in the therapeutic choice. However, the most important efficacy outcomes for physicians were clinical remission and endoscopic response, while the main interest for patients were the achievement of clinical response or clinical remission. These data are in line with the literature, confirming that the absence of symptoms is the main objective of patients [8,9]. While this patient preference is understandable since IBD are chronic diseases that greatly impact patients’ quality of life; on the other hand, it underlines the need to provide more information to patients. Patient engagement in treatment decisions has been associated with improved quality of life and may be associated with greater well-being and improved outcomes [10,11]. Presence of comorbidities and disease activity were essential elements of the therapeutic decision in both study groups. However, while physicians prioritized the previous failure of other medications, patients’ primary concern was to improve quality of life. Surprisingly, disease duration, dosing interval, need to go to hospital, and response time to drug were relevant factors in treatment choice for most physicians, but they had a limited role for a remarkable proportion of patients. This discrepancy suggests that physicians and patients often have different points of view and a different knowledge. Interestingly, the acceptable response time to therapy was also different in the two groups, with most patients reporting a shorter response time than physicians (<4 weeks vs. <3 months, respectively). IBD therapies have an induction and a maintenance phase. Generally, the first assessment of disease activity is performed at the end of the induction phase, thus explaining the timing considered appropriate by most clinicians [12]. On the other hand, patients would prefer an improvement in the shortest possible time. In view of this important patient demand, more and more attention is paid to the response time. The new small molecules are extremely rapid drugs that have been shown to achieve improvement after just one day of therapy [13,14,15,16]. Unfortunately, it is not possible to predict the response time to therapies. Although there are available therapeutic options, a considerable proportion of patients do not have a well-controlled disease and require surgery [17]. For this reason, it is advisable to involve patients in therapeutic decisions, informing them of any critical issues and symptoms to be monitored (e.g., number of evacuations, bleeding, and severe abdominal pain) in order to set up a personalized and tailored approach. Regarding the safety profile, patients and physicians agreed on the need to evaluate the risk of infections, severe infections, malignancy, cardiovascular events, hospitalization, surgery, and death, but the risk percentages considered acceptable by the two groups differed. Surprisingly, an annual infectious risk of up to 10% was reported by physicians, while a relevant proportion of patients (about 20%) accepted higher risks (25% and 50% per year). A recent systematic review and meta-analysis including randomized clinical trials investigated the infection risk of available biological drugs in CD (anti-TNF agents, vedolizumab and ustekinumab) [18]. None of the evaluated biologics (except for adalimumab) had an increased risk of infections compared with placebo, confirming their reliable safety profile. Similarly, in a systematic review and meta-analysis including 15 studies (9 observational cohort studies, 5 case-control studies, and 1 post hoc analysis of a randomized trial) no increased risk of infections or malignancy was found in elderly patients with IBD exposed to biologics [19]. On the other hand, vedolizumab in UC and ustekinumab in CD were associated with a lower risk of severe infections in patients with IBD, suggesting that a careful assessment of the patients’ safety profile should be performed before choosing a therapy [20]. Of note, some patients reported that a 5-year risk of death of up to 50% was acceptable. This data suggests the negative impact of an active disease on quality of life, and even if extreme, it shows that some subjects are theoretically willing to accept a negative prognosis in order to quickly alleviate the symptoms of the disease. In both CD and UC, infliximab was considered the most effective drug for inducing and maintaining disease remission, followed by vedolizumab in UC and ustekinumab in CD. These results are in line with the literature, demonstrating adequate knowledge of survey participants. Indeed, a systematic review and network meta-analysis of phase 2 and phase 3 randomized controlled trials confirmed that infliximab was the most effective drug in inducing clinical remission in CD and suggested the use of anti-TNF agents as first line therapy and inhibitors of interleukin 23 pathway, including ustekinumab, as second line [18]. Similarly, a systematic review and meta-analysis evaluated the efficacy of biological drugs and small molecules in UC phase III trials controlled with placebo or active comparator [21]. Upadacitinib was the most effective drug in inducing clinical remission while infliximab was ranked the highest in achieving endoscopic improvement. Another meta-analysis of randomized controlled trials by Lasa and colleagues confirmed upadacitinib as the most effective drug in inducing clinical remission in UC followed by infliximab [22]. Vedolizumab, on the other hand, was associated with the lowest risk of adverse events and serious adverse events. Despite the effectiveness of the available drugs, a significant percentage of patients do not respond or lose response to therapy over time underlining the unmet need for new strategies [23,24,25,26]. Growing evidence shows that the combination of biologics and/or small molecules is a viable therapeutic option in IBD [27,28,29]. However, it remains to be defined which patients are eligible for this approach [30]. The risk-benefit assessment of a therapy should be calibrated on the need for a more robust treatment, such as a dual therapy. A global survey of the International Organization for the Study of IBD (IOIBD) investigated the characteristics of difficult-to-treat patients [31]. The number of previously failed drugs and drug classes, need for surgery, concomitant presence of primary sclerosing cholangitis or perianal disease were identified as key elements in defining difficult-to-treat patients. An IOIBD initiative is ongoing to provide a validated definition of difficult-to-treat patients. It will then be necessary to conduct studies to evaluate whether the early use of dual therapy in this challenging scenario could be considered a valid option.

Our survey has several strengths. To the best of our knowledge, this is the first survey specifically designed to investigate the risk-benefit ratio before starting an IBD therapy. Our work provides very useful insights for understanding the opinions of health care providers and patients and could represent the starting point for the development of an easy-to-use tool in order to standardize the benefit-risk assessment of a new drug for IBD. Moreover, a large number of patients and physicians from different countries around the world were included, making the data more reliable. However, there are also limitations. In fact, the questionnaire used was not validated and the number of patients included for each center was not balanced, preventing any differences between the different countries from being evaluated.

## 5. Conclusions

Patients and physicians have different perspectives on the risk-benefit ratio of a therapy. The constant scientific updating of medical doctors and adequate information and sharing with patients are key aspects for optimal management of inflammatory bowel diseases. There is an urgent need to develop a validated and commonly used tool for both clinical practice and randomized clinical trials to assess the risk-benefit ratio of a new treatment in order to standardize therapeutic decisions and improve disease control.

## Figures and Tables

**Table 1:** Patients’ demographics.

	Number (%)
Median age	40 (min 17–max 82) years
Male	163/367 (44.4%)
Crohn’s disease	221 (60.2%)
Ulcerative colitis	144 (39.2%)
Unclassified-IBD	2 (0.6%)
Median age at diagnosis	26 (min 6–max 77) years
Steroids	256 (69.7%)
Immunosuppressants	163 (44.4%)
Biological drugs and small molecules	298 (81.2%)

**Table 2:** Physicians’ demographics.

	Number (%)
Median age	51 (min 29–max 82) years
Male	78/146 (53.4%)
Gastroenterologists	129 (88.4%)
Internal Doctors	6 (4.1%)
Surgeons	5 (3.4%)
Other	5 (3.4%)
General practitioners	1 (0.7%)
Years of experience in the field of IBD	
>10 years	105 (71.9%)
>5–10 years	25 (17.1%)
1–5 years	13 (8.9%)
<1 year	3 (2.1%)
IBD patients visited per year	
<100 patients	70 (48.0%)
100–500 patients	41 (28.1%)
500–1000 patients	24 (16.4%)
>1000 patients	11 (7.5%)

## Data Availability

The data underlying this article will be shared on reasonable request to the corresponding author.

## Supplementary Materials

The following supporting information can be downloaded at: https://www.mdpi.com/article/10.3390/jcm12093094/s1, Supplementary File S1: Survey for patients; Supplementary File S2: Survey for physicians; Supplementary File S3: Number of participating physicians for each country.
