# Supplementary material for: Risk-Benefit of IBD Drugs: A Physicians and Patients Survey
Source: J Clin Med. 2023 Apr 24;12(9):3094. doi: 10.3390/jcm12093094 (PMC10179123; doi:10.3390/jcm12093094)
Supplement: Supplementary file 1 [file jcm-12-03094-s001.zip › jcm-2344057-supplementary.pdf]

Supplementary file S1: Survey for patients

1) Age in years

2) Sex
a) Male
b) Female

3) What country do you live in?

4) What is your inflammatory bowel disease?
a) Crohn's disease
b) Ulcerative colitis
c) Unclassified colitis

5) Age at diagnosis?

6) Have you ever been treated with steroids?
a) Yes
b) No
c) Do not know

7) Have you ever been treated with immunosuppressive drugs (e.g. thiopurines or methotrexate)?
a) Yes
b) No
c) Do not know

8) Have you ever been treated with biological drugs or small molecules (e.g. infliximab, adalimumab, vedolizumab, ustekinumab, or tofacitinib)?
a) Yes
b) No
c) Do not know

9) In the benefit-risk evaluation before starting a new therapy, what is the most important factor to consider?
a) Efficacy
b) Safety
c) Equally efficacy and safety
d) Do not know

10) How would you rate the relevance of drug efficacy in evaluating the benefit-risk ratio of IBD therapy using a scale from 0 to 100 (0= not relevant at all, 100 = very relevant)?

11) Do you think achieving clinical improvement is important in evaluating the benefit of IBD therapy?
a) Yes
b) No

c) Do not know

12) How would you rate the importance of achieving clinical improvement in evaluating the benefit of IBD therapy using a scale from 0 to 100 (0= not important at all, 100 = very important)?

13) Do you think achieving clinical remission is important in evaluating the benefit of IBD therapy?

a) Yes
b) No
c) Do not know

14) How would you rate the importance of achieving clinical remission in evaluating the benefit of IBD therapy using a scale from 0 to 100 (0= not important at all, 100 = very important)?

15) Do you think achieving endoscopic improvement is important in evaluating the benefit of IBD therapy?

a) Yes
b) No
c) Do not know

16) How would you rate the importance of achieving endoscopic improvement in evaluating the benefit of IBD therapy using a scale from 0 to 100 (0= not important at all, 100 = very important)?

17) Do you think achieving endoscopic remission is important in evaluating the benefit of IBD therapy?

a) Yes
b) No
c) Do not know

18) How would you rate the importance of achieving endoscopic remission in evaluating the benefit of IBD therapy using a scale from 0 to 100 (0= not important at all, 100 = very important)?

19) Do you think achieving biochemical remission (e.g. normalization in CRP and fecal calprotectin levels) is important in evaluating the benefit of IBD therapy?

a) Yes
b) No
c) Do not know

20) How would you rate the importance of achieving biochemical remission in evaluating the benefit of IBD therapy using a scale from 0 to 100 (0= not important at all, 100 = very important)?

21) Do you think achieving histological remission is important in evaluating the benefit of UC therapy?

a) Yes
b) No
c) Do not know

22) How would you rate the importance of histological remission in evaluating the benefit of UC therapy using a scale from 0 to 100 (0= not important at all, 100 = very important)?

23) Do you think achieving radiological remission is important in evaluating the benefit of CD therapy?

a) Yes
b) No
c) Do not know

24) How would you rate the importance of radiological remission in evaluating the benefit of CD therapy using a scale from 0 to 100 (0= not important at all, 100 = very important)?

25) Is the response time to therapy a relevant factor for the therapeutic choice of a drug?

a) Yes
b) No
c) Do not know

26) If you answered yes to the previous question, how soon should the drug be effective to justify its use?

a) Within 1 week
b) Within 2 weeks
c) Within 4 weeks
d) Within 3 months
e) Do not know

27) Should the benefit-risk ratio of a drug take into consideration the age of the patient?

a) Yes
b) No
c) Do not know

28) Should the benefit-risk ratio of a drug take into consideration the activity of disease (e.g. mild, moderate, or severe disease)?

a) Yes
b) No
c) Do not know

29) Should the extension of disease be considered in the risk-benefit ratio?

a) Yes
b) No
c) Do not know

30) Should the duration of disease be considered in the risk-benefit ratio?

a) Yes
b) No
c) Do not know

31) Should the presence of comorbidities (e.g. rheumatological, cardiovascular, or dermatological diseases) be considered in the risk-benefit ratio?

a) Yes

b) No
c) Do not know

32) Should family history of IBD be considered in the risk-benefit ratio?
a) Yes
b) No
c) Do not know

33) Should the administration route of a drug (e.g. intravenous, subcutaneous, or oral) be considered in the risk-benefit ratio?
a) Yes
b) No
c) Do not know

34) Should the administration interval (e.g. every day, every week, every 2 weeks etc) be considered in the treatment decision?
a) Yes
b) No
c) Do not know

35) Should the therapy duration be considered in the risk-benefit ratio of initiating IBD therapy?
a) Yes
b) No
c) Do not know

36) Should the patients' quality of life be considered in the risk-benefit ratio of initiating IBD therapy?
a) Yes
b) No
c) Do not know

37) Should the need to go to hospital for treatment be considered in the risk-benefit ratio (including loss of working days and need for a caregiver)?
a) Yes
b) No
c) Do not know

38) Should prior failure towards biologics or small molecules be included in the benefit-risk assessment of an IBD drug?
a) Yes
b) No
c) Do not know

39) How would you rate the relevance of drug safety in evaluating the benefit-risk ratio of IBD therapy using a scale from 0 to 100 (0= not relevant at all, 100 = very relevant)?

40) Should the risk of infection be included in the benefit-risk assessment of an IBD patient who is a candidate for therapy?

a) Yes
b) No
c) Do not know

41) If you answered yes to the previous question, what is the acceptable risk of infections?
a) No risk
b) less than 1% per year
c) less than 5% per year
d) less than 10% per year
e) less than 25% per year
f) less than 50% per year
g) Do not know

42) Should the risk of serious infection be included in the benefit-risk assessment of an IBD patient who is a candidate for therapy?
a) Yes
b) No
c) Do not know

43) If you answered yes to the previous question, what is the acceptable risk of serious infections?
a) No risk
b) less than 1% per year
c) less than 5% per year
d) less than 10% per year
e) less than 25% per year
f) less than 50% per year
g) Do not know

44) Should the risk of malignancy be included in the benefit-risk assessment of an IBD patient who is a candidate for therapy?
a) Yes
b) No
c) Do not know

45) If you answered yes to the previous question, what is the acceptable risk of malignancies?
a) No risk
b) less than 1% at 5 years
c) less than 5% at 5 years
d) less than 10% at 5 years
e) less than 25% at 5 years
f) less than 50% at 5 years
g) Do not know

46) Should the risk of cardiovascular events (e.g. thrombosis, pulmonary embolism) be included in the benefit-risk assessment of an IBD patient who is a candidate for therapy?
a) Yes
b) No
c) Do not know

47) If you answered yes to the previous question, what is the acceptable risk of cardiovascular events?

a) No risk
b) less than 1% per year
c) less than 5% per year
d) less than 10% per year
e) less than 25% per year
f) less than 50% per year
g) Do not know

48) Should the risk of disease relapse be included in the benefit-risk assessment of an IBD patient who is a candidate for therapy?

a) Yes
b) No
c) Do not know

49) If you answered yes to the previous question, what is the acceptable risk of relapse?

a) No risk
b) less than 1% per year
c) less than 5% per year
d) less than 10% per year
e) less than 25% per year
f) less than 50% per year
g) Do not know

50) Should the risk of surgery after initiation of therapy be considered in the benefit-risk assessment of an IBD patient who is a candidate for therapy?

a) Yes
b) No
c) Do not know

51) If you answered yes to the previous question, what is the acceptable risk of surgery?

a) No risk
b) less than 1% at 5 years
c) less than 5% at 5 years
d) less than 10% at 5 years
e) less than 25% at 5 years
f) less than 50% at 5 years
g) Do not know

52) Should the risk of hospitalization after initiation of therapy be considered in the benefit-risk assessment of an IBD patient who is a candidate for therapy?

a) Yes
b) No
c) Do not know

53) If you answered yes to the previous question, what is the acceptable risk of hospitalization?

a) No risk

b)	less than 1% at 5 years
c)	less than 5% at 5 years
d)	less than 10% at 5 years
e)	less than 25% at 5 years
f)	less than 50% at 5 years
g)	Do not know

54)	Should the risk of death after initiation of therapy be considered in the benefit-risk assessment of an IBD patient who is a candidate for therapy?
a) Yes
b) No
c) Do not know

55)	If you answered yes to the previous question, what is the acceptable risk of death?
h)	No risk
i)	less than 1% at 5 years
j)	less than 5% at 5 years
k)	less than 10% at 5 years
l)	less than 25% at 5 years
m)	less than 50% at 5 years
n)	Do not know

56)	In specific populations at risk of adverse events (e.g. pediatric patients, pregnant women, the elderly, personal cancer history) should safety be the main feature of the drug?
a)	Yes
b)	No
c)	Do not know

Supplementary file S2: Survey for physicians

57) Age in years

58) Sex
c) Male
d) Female

59) What country do you work in?

60) What is your specialization?
a) Gastroenterologist
b) Internal doctor
c) Surgeon
d) General practitioner
e) Other (Please specify)

61) How many years of experience do you have in the field of IBD?
a) Less than 1 year
b) Less than 5 years
c) Less than 10 years
d) More than 10 years

62) How many IBD patients do you visit in 1 year?
a) Less than 100
b) Less than 500
c) Less than 1000
d) More than 1000

63) In the benefit-risk evaluation before starting a new therapy, what is the most important factor to consider?
e) Efficacy
f) Safety
g) Efficacy and safety equally
h) Do not know

64) How would you rate the relevance of drug efficacy in evaluating the benefit-risk ratio of IBD therapy using a scale from 0 to 100 (0= not relevant at all, 100 = very relevant)?

65) Do you think achieving clinical response is important in evaluating the benefit of IBD therapy?
d) Yes
e) No
f) Do not know

66) How would you rate the importance of achieving clinical response in evaluating the benefit of IBD therapy using a scale from 0 to 100 (0= not important at all, 100 = very important)?

67) Do you think achieving clinical remission is important in evaluating the benefit of IBD therapy?
d) Yes
e) No
f) Do not know

68) How would you rate the importance of achieving clinical remission in evaluating the benefit of IBD therapy using a scale from 0 to 100 (0= not important at all, 100 = very important)?

69) Do you think achieving endoscopic response is important in evaluating the benefit of IBD therapy?

d) Yes
e) No
f) Do not know

70) How would you rate the importance of achieving endoscopic response in evaluating the benefit of IBD therapy using a scale from 0 to 100 (0= not important at all, 100 = very important)?

71) Do you think achieving endoscopic remission is important in evaluating the benefit of IBD therapy?
d) Yes
e) No
f) Do not know

72) How would you rate the importance of achieving endoscopic remission in evaluating the benefit of IBD therapy using a scale from 0 to 100 (0= not important at all, 100 = very important)?

73) Do you think achieving biochemical remission (e.g. CRP and fecal calprotectin) is important in evaluating the benefit of IBD therapy?
d) Yes
e) No
f) Do not know

74) How would you rate the importance of achieving biochemical remission in evaluating the benefit of IBD therapy using a scale from 0 to 100 (0= not important at all, 100 = very important)?

75) Do you think achieving histological remission is important in evaluating the benefit of UC therapy?
d) Yes
e) No
f) Do not know

76) How would you rate the importance of histological remission in evaluating the benefit of UC therapy using a scale from 0 to 100 (0= not important at all, 100 = very important)?

77) Do you think achieving radiological remission is important in evaluating the benefit of CD therapy?
d) Yes

e) No
f) Do not know

78) How would you rate the importance of radiological remission in evaluating the benefit of CD therapy using a scale from 0 to 100 (0= not important at all, 100 = very important)?

79) Is the response time to therapy a relevant factor for the therapeutic choice of a drug?
d) Yes
e) No
f) Do not know

80) If you answered yes to the previous question, how soon should the drug be effective to justify its use?
f) Within 1 week
g) Within 2 weeks
h) Within 4 weeks
i) Within 3 months
j) Do not know

81) Should the benefit-risk ratio of a drug take into consideration the age of the patient?
d) Yes
e) No
f) Do not know

82) Should the benefit-risk ratio of a drug take into consideration the activity of disease (e.g. mild, moderate, or severe disease)?
d) Yes
e) No
f) Do not know

83) Should the extension of disease be considered in the risk-benefit ratio?
d) Yes
e) No
f) Do not know

84) Should the duration of disease be considered in the risk-benefit ratio?
d) Yes
e) No
f) Do not know

85) Should the presence of comorbidities (e.g. rheumatological, cardiovascular, or dermatological diseases) be considered in the risk-benefit ratio?
d) Yes
e) No
f) Do not know

86) Should family history of IBD be considered in the risk-benefit ratio?
d) Yes
e) No

f) Do not know

87) Should the administration route of a drug (e.g. intravenous, subcutaneous, or oral) be considered in the risk-benefit ratio?

d) Yes
e) No
f) Do not know

88) Should the administration interval (e.g. every day, every week, every 2 weeks etc) be considered in the treatment decision?

d) Yes
e) No
f) Do not know

89) Should the therapy duration be considered in the risk-benefit ratio of initiating IBD therapy?

d) Yes
e) No
f) Do not know

90) Should the patients' quality of life be considered in the risk-benefit ratio of initiating IBD therapy?

d) Yes
e) No
f) Do not know

91) Should the need to go to hospital for treatment be considered in the risk-benefit ratio (including loss of working days and need for a caregiver)?

d) Yes
e) No
f) Do not know

92) Should prior failure towards biologics or small molecules be included in the benefit-risk assessment of an IBD drug?

d) Yes
e) No
f) Do not know

93) How would you rate the relevance of drug safety in evaluating the benefit-risk ratio of IBD therapy using a scale from 0 to 100 (0= not relevant at all, 100 = very relevant)?

94) Should the risk of infection be included in the benefit-risk assessment of an IBD patient who is a candidate for therapy?

d) Yes
e) No
f) Do not know

95) If you answered yes to the previous question, what is the acceptable risk of infections?

h) No risk

i) less than 1% per year
j) less than 5% per year
k) less than 10% per year
l) less than 25% per year
m) less than 50% per year
n) Do not know

96) Should the risk of serious infection be included in the benefit-risk assessment of an IBD patient who is a candidate for therapy?
d) Yes
e) No
f) Do not know

97) If you answered yes to the previous question, what is the acceptable risk of serious infections?
h) No risk
i) less than 1% per year
j) less than 5% per year
k) less than 10% per year
l) less than 25% per year
m) less than 50% per year
n) Do not know

98) Should the risk of malignancy be included in the benefit-risk assessment of an IBD patient who is a candidate for therapy?
d) Yes
e) No
f) Do not know

99) If you answered yes to the previous question, what is the acceptable risk of malignancies?
h) No risk
i) less than 1% at 5 years
j) less than 5% at 5 years
k) less than 10% at 5 years
l) less than 25% at 5 years
m) less than 50% at 5 years
n) Do not know

100) Should the risk of cardiovascular events (e.g. thrombosis, pulmonary embolism) be included in the benefit-risk assessment of an IBD patient who is a candidate for therapy?
d) Yes
e) No
f) Do not know

101) If you answered yes to the previous question, what is the acceptable risk of cardiovascular events?
h) No risk
i) less than 1% per year
j) less than 5% per year

k) less than 10% per year
l) less than 25% per year
m) less than 50% per year
n) Do not know

102) Should the risk of disease relapse be included in the benefit-risk assessment of an IBD patient who is a candidate for therapy?
c) Yes
d) No
c) Do not know

103) If you answered yes to the previous question, what is the acceptable risk of relapse?
h) No risk
i) less than 1% per year
j) less than 5% per year
k) less than 10% per year
l) less than 25% per year
m) less than 50% per year
n) Do not know

104) Should the risk of surgery after initiation of therapy be considered in the benefit-risk assessment of an IBD patient who is a candidate for therapy?
d) Yes
e) No
f) Do not know

105) If you answered yes to the previous question, what is the acceptable risk of surgery?
h) No risk
i) less than 1% at 5 years
j) less than 5% at 5 years
k) less than 10% at 5 years
l) less than 25% at 5 years
m) less than 50% at 5 years
n) Do not know

106) Should the risk of hospitalization after initiation of therapy be considered in the benefit-risk assessment of an IBD patient who is a candidate for therapy?
d) Yes
e) No
f) Do not know

107) If you answered yes to the previous question, what is the acceptable risk of hospitalization?
o) No risk
p) less than 1% at 5 years
q) less than 5% at 5 years
r) less than 10% at 5 years
s) less than 25% at 5 years
t) less than 50% at 5 years
u) Do not know

108) Should the risk of death after initiation of therapy be considered in the benefit-risk assessment of an IBD patient who is a candidate for therapy?

d) Yes
e) No
f) Do not know

109) If you answered yes to the previous question, what is the acceptable risk of death?

v) No risk
w) less than 1% at 5 years
x) less than 5% at 5 years
y) less than 10% at 5 years
z) less than 25% at 5 years
aa) less than 50% at 5 years
bb) Do not know

110) In specific populations at risk of adverse events (e.g. pediatric patients, pregnant women, the elderly, personal cancer history) should safety be the main feature of the drug?

d) Yes
e) No
f) Do not know

111) How would you rate the efficacy of thiopurines in inducing remission in ulcerative colitis using a scale from 0 to 100 (0 = not effective at all, 100 = very effective)?

112) How would you rate the efficacy of thiopurines in inducing remission in Crohn's disease using a scale from 0 to 100 (0 = not effective at all, 100 = very effective)?

113) How would you rate the efficacy of thiopurines in maintaining remission in ulcerative colitis using a scale from 0 to 100 (0 = not effective at all, 100 = very effective)?

114) How would you rate the efficacy of thiopurines in maintaining remission in Crohn's disease using a scale from 0 to 100 (0 = not effective at all, 100 = very effective)?

115) How would you rate the overall safety of thiopurines in the treatment of Crohn's disease and ulcerative colitis using a scale from 0 to 100 (0 = not safe at all, 100 = very safe)?

116) How would you rate the efficacy of methotrexate in inducing remission in ulcerative colitis using a scale from 0 to 100 (0 = not effective at all, 100 = very effective)?

117) How would you rate the efficacy of methotrexate in inducing remission in Crohn's disease using a scale from 0 to 100 (0 = not effective at all, 100 = very effective)?

118) How would you rate the efficacy of methotrexate in maintaining remission in ulcerative colitis using a scale from 0 to 100 (0 = not effective at all, 100 = very effective)?

119) How would you rate the efficacy of methotrexate in maintaining remission in Crohn's disease using a scale from 0 to 100 (0 = not effective at all, 100 = very effective)?

120) How would you rate the overall safety of methotrexate in the treatment of Crohn's disease and ulcerative colitis using a scale from 0 to 100 (0 = not safe at all, 100 = very safe)?

121) How would you rate the efficacy of adalimumab in inducing remission in ulcerative colitis using a scale from 0 to 100 (0 = not effective at all, 100 = very effective)?

122) How would you rate the efficacy of adalimumab in inducing remission in Crohn's disease using a scale from 0 to 100 (0 = not effective at all, 100 = very effective)?

123) How would you rate the efficacy of adalimumab in maintaining remission in ulcerative colitis using a scale from 0 to 100 (0 = not effective at all, 100 = very effective)?

124) How would you rate the efficacy of adalimumab in maintaining remission in Crohn's disease using a scale from 0 to 100 (0 = not effective at all, 100 = very effective)?

125) How would you rate the overall safety of adalimumab in the treatment of Crohn's disease and ulcerative colitis using a scale from 0 to 100 (0= not safe at all, 100 = very safe)?

126) How would you rate the efficacy of infliximab in inducing remission in ulcerative colitis using a scale from 0 to 100 (0 = not effective at all, 100 = very effective)?

127) How would you rate the efficacy of infliximab in inducing remission in Crohn's disease using a scale from 0 to 100 (0 = not effective at all, 100 = very effective)?

128) How would you rate the efficacy of infliximab in maintaining remission in ulcerative colitis using a scale from 0 to 100 (0 = not effective at all, 100 = very effective)?

129) How would you rate the efficacy of infliximab in maintaining remission in Crohn's disease using a scale from 0 to 100 (0 = not effective at all, 100 = very effective)?

130) How would you rate the overall safety of infliximab in the treatment of Crohn's disease and ulcerative colitis using a scale from 0 to 100 (0= not safe at all, 100 = very safe)?

131) How would you rate the efficacy of golimumab in inducing remission in ulcerative colitis using a scale from 0 to 100 (0 = not effective at all, 100 = very effective)?

132) How would you rate the efficacy of golimumab in maintaining remission in ulcerative colitis using a scale from 0 to 100 (0 = not effective at all, 100 = very effective)?

133) How would you rate the overall safety of golimumab in the treatment of ulcerative colitis using a scale from 0 to 100 (0 = not safe at all, 10 = very safe)?

134) How would you rate the efficacy of vedolizumab in inducing remission in ulcerative colitis using a scale from 0 to 100 (0= not effective at all, 100 = very effective)?

135) How would you rate the efficacy of vedolizumab in inducing remission in Crohn's disease using a scale from 0 to 100 (0 = not effective at all, 100 = very effective)?

136) How would you rate the efficacy of vedolizumab in maintaining remission in ulcerative colitis using a scale from 0 to 100 (0= not effective at all, 100 = very effective)?

137) How would you rate the efficacy of vedolizumab in maintaining remission in Crohn's disease using a scale from 0 to 100 (0 = not effective at all, 100 = very effective)?

138) How would you rate the overall safety of vedolizumab in the treatment of Crohn's disease and ulcerative colitis using a scale from 0 to 100 (0 = not safe at all, 100 = very safe)?

139) How would you rate the efficacy of ustekinumab in inducing remission in ulcerative colitis using a scale from 0 to 100 (0= not effective at all, 100 = very effective)?

140) How would you rate the efficacy of ustekinumab in inducing remission in Crohn's disease using a scale from 0 to 100 (0 = not effective at all, 100 = very effective)?

141) How would you rate the efficacy of ustekinumab in maintaining remission in ulcerative colitis using a scale from 0 to 100 (0 = not effective at all, 100 = very effective)?

142) How would you rate the efficacy of ustekinumab in maintaining remission in Crohn's disease using a scale from 0 to 100 (0 = not effective at all, 100 = very effective)?

143) How would you rate the overall safety of ustekinumab in the treatment of Crohn's disease and ulcerative colitis using a scale from 0 to 100 (0 = not safe at all, 100 = very safe)?

144) How would you rate the efficacy of tofacitinib in inducing remission in ulcerative colitis using a scale from 0 to 100 (0 = not effective at all, 100 = very effective)?

145) How would you rate the efficacy of tofacitinib in maintaining remission in ulcerative colitis using a scale from 0 to 100 (0 = not effective at all, 100 = very effective)?

146) How would you rate the overall safety of tofacitinib in the treatment of ulcerative colitis using a scale from 0 to 100 (0 = not safe at all, 100 = very safe)?

Supplementary file S3: Number of participating physicians for each country

	number (%)
Italy	22 (15.1%)
Brazil	8 (5.5%)
Lebanon	8 (5.5%)
Russia	7 (4.8%)
Australia	6 (4.1%)
Greece	6 (4.1%)
India	5 (3.4%)
Spain	5 (3.4%)
Algeria	4 (2.7%)
Serbia	4 (2.7%)
Belarus	3 (2.1%)
Belgium	3 (2.1%)
Chile	3 (2.1%)
Iran	3 (2.1%)
Slovenia	3 (2.1%)
South Africa	3 (2.1%)
United Kingdom	3 (2.1%)
Vietnam	3 (2.1%)
Austria	2 (1.4%)
Bangladesh	2 (1.4%)
Croatia	2 (1.4%)
Egypt	2 (1.4%)
France	2 (1.4%)
Hungary	2 (1.4%)
Israel	2 (1.4%)
Kazakhstan	2 (1.4%)
Mexico	2 (1.4%)
Portugal	2 (1.4%)
Romania	2 (1.4%)
Saudi Arabia	2 (1.4%)
Slovakia	2 (1.4%)
Switzerland	2 (1.4%)
United Arab Emirates	2 (1.4%)
Argentina	1 (0.7%)
Bosnia	1 (0.7%)
Canada	1 (0.7%)
China	1 (0.7%)
Denmark	1 (0.7%)
Ecuador	1 (0.7%)
Honduras	1 (0.7%)
Ireland	1 (0.7%)
Jamaica	1 (0.7%)
Lithuania	1 (0.7%)
New Zealand	1 (0.7%)
Northern Ireland	1 (0.7%)
Peru	1 (0.7%)
Singapore	1 (0.7%)
Sweden	1 (0.7%)
United States	1 (0.7%)
Uruguay	1 (0.7%)
